# Cross-sectional association of equol producing status with aortic calcification in Japanese men aged 40–79 years

**DOI:** 10.1038/s41598-022-24659-8

**Published:** 2022-11-22

**Authors:** Xiao Zhang, Akira Fujiyoshi, Aya Kadota, Keiko Kondo, Sayuki Torii, Yukiko Okami, Takashi Hisamatsu, Yuichiro Yano, Emma Barinas-Mitchell, Jared Magnani, Katsuyuki Miura, Hirotsugu Ueshima, Akira Sekikawa

**Affiliations:** 1 Department of Epidemiology, School of Public Health, University of Pittsburgh, 130 North Bellefield Avenue, Suite 331, Pittsburgh, PA 15213 USA; 2 Vanke School of Public Health, Tsinghua University, Beijing, China; 3 Department of Hygiene, Wakayama Medical University, Wakayama, Japan; 4 Department of Public Health, Shiga University of Medical Science, Otsu, Japan; 5 Department of Public Health, Okayama University Graduate School of Medicine, Dentistry and Pharmaceutical Sciences, Okayama, Japan; 6 NCD Epidemiology Research Center, Shiga University of Medical Science, Otsu, Japan; 7 Department of Medicine, University of Pittsburgh, Pittsburgh, USA

**Keywords:** Nutrition, Nutritional supplements, Epidemiology, Epidemiology, Calcification, Calcification, Aortic diseases, Atherosclerosis, Calcification

## Abstract

Equol is an isoflavone (ISF)-derived metabolite by the gut microbiome in certain individuals termed equol-producers (EP). Equol might be the critical anti-atherogenic component of ISFs. In a population-based study of 979 Japanese men aged 40–79 without cardiovascular (CVD) or chronic kidney disease, we measured the urinary levels of equol and ISFs. Aortic calcification (AC) in the entire aorta was assessed by electron-beam or multi-detector-row computed tomography. Subjects with log10 (urinary equol to daidzein concentration) > − 1.5 were classified as EP. EP was further classified as person with low- and high-equol. We analyzed the association between equol-producing status and AC presence, defined as AC score > 0, by the logistic regressions. We found that EP (50% of the sample) had significantly lower odds of AC presence (odds ratio (OR): 0.62, 95% confidence interval (CI): 0.39, 0.98) compared to non-EP. This association was independent of CVD risk factors. For the dose–response association, compared to non-EP, subjects with low and high levels of equol had ORs of 0.51 (95% CI 0.30, 0.84) and 0.67 (95% CI 0.39, 1.14) after adjusting for major CVD risk factors (*p* for trend = 0.06). ISFs concentrations were not significantly associated with AC presence (OR: 1.18, 95% CI: 0.82, 1.69). In conclusion, EP had a significantly lower burden of AC than non-EP, while ISFs were not associated with AC presence in Japanese men aged 40–79 years.

## Introduction

Soy isoflavones (ISFs) are non-steroidal phytoestrogens regularly consumed in East Asian countries. In contrast, their intake in the US is minimal (an average of 25–50 mg/day in East Asia vs. < 2 mg/day in the US^1–3^). Estradiol exerts its biological action by binding both estrogen receptor α (ERα) expressed in the reproductive, central nervous, cardiovascular, and other systems and estrogen receptor β (ERβ) expressed in the cardiovascular, central nervous, and other systems. ISFs, however, preferentially bind to ERβ^4,5^. Several studies in East Asian countries reported that dietary intake of ISFs is significantly and inversely associated with incident coronary heart disease (CHD)^6,7^. However, a randomized clinical trial in the US among 350 postmenopausal women showed that ISFs intervention for 2.7 years had a null treatment effect on atherosclerosis overall^8^. This discrepancy may be due to the higher capacity of producing equol after consuming ISFs among people living in the East Asian countries than in the US; such individuals are referred to as “equol-producers (EP)”^9^. Equol is a metabolite of an ISF daidzein by the gut bacteria^10^. The encoding of such bacterial strains involves enzymes^11–13^ and daidzein can be metabolized to equol via several reactions, including reduction, methylation, demethylation, hydroxylation, dihydroxylation and C-ring cleavage. Compared to ISFs, equol is more biologically active, a more potent antioxidant, and has a similar or greater affinity to ERβ and thus may have higher anti-atherogenic properties^10,14^. A nested case–control study within a prospective cohort study in China demonstrated that urinary equol but not ISFs or their other metabolites was significantly inversely associated with incident CHD^6^. Interestingly, 50 to 70% of the population residing in East Asian countries are equol-producers compared to 20 to 30% in Western countries. This is due to the differences in the microbiome but not genetics^10^ and suggests that equol may be the critical anti-atherogenic component of ISFs.

Aortic calcification (AC) and coronary artery calcification (CAC) are biomarkers of atherosclerosis. While CAC is a powerful predictor of future cardiovascular events and significantly improves the classification of cardiovascular risk status^15^, several studies have suggested that both presence and progression of AC significantly predict future cardiovascular events independent of CAC^16–18^. We have recently reported that EP had a lower AC prevalence than non-EP among 302 men in Japan aged 40–49^19^. However, the association was not statistically significant due to a relatively small number of middle-aged men. This study aimed to assess the association between equol producing status and AC in population-based Japanese men aged 40–79 years old from the Shiga Epidemiological Study of Subclinical Atherosclerosis (SESSA) study. SESSA was designed to understand cardiovascular risks among Japanese and US populations. We hypothesize that EP had a significantly lower burden of AC than non-EP.

## Results

Among the 979 participants, 50.3% (n = 492) were EP. The characteristics of EP and non-EP (n = 487) are described in Table 1. EP had significantly higher age and lower concentration of creatine-adjusted urinary daidzein and genistein than non-EP. Moreover, EP had a higher chance of no AC presence than non-EP. The proportion of participants having CVD risk factors was similar between EP and non-EP.

**Table 1:** Characteristics of analysis sample by equol producing status (n = 979).

		Equol-producer (n = 492)	Non-equol-producer (n = 487)	*p*-value
Age (year)		65.0 (9.6)	62.4 (10.0)	< 0.001
Pack-year of smoking		24 (3.3–44.0)	25 (6–43.5)	0.41
Ethanol consumption (gram/week)		98 (7–252)	98 (5.9–259.3)	0.59
eGFR (ml/min/1.73 m^2^)		74.4 (9.4)	75.3 (9.8)	0.15
Creatine-adjusted urinary daidzein		3846 (1312–11,230)	8370 (2957–23,543)	< 0.001
Creatine-adjusted urinary genistein		3479 (1315–8215)	5010 (1842–13,421)	< 0.001
Aortic calcification score (Agatston unit)	0%	0	0	0.004
	25%	46	23	
	50%	381	259	
	75%	1549	1021	
	100%	20,007	13,463	
Aortic calcification category	0	83 (17%)	79 (16%)	0.01
	1–99	71 (14%)	109 (23%)	
	100–299	73 (15%)	66 (14%)	
	300–999	96 (20%)	110 (23%)	
	≥ 1000	169 (34%)	123 (25%)	
Hypertension		270 (55%)	258 (53%)	0.55
Diabetes		99 (20%)	104 (21%)	0.75
Hypercholesterolemia		221 (40%)	194 (36%)	0.18
Obesity		129 (26%)	153 (31%)	0.07

Mean (standard deviation) is shown if the distribution of a continuous variable is normal; median (25th–75th percentile) is shown if the distribution of a continuous variable is skewed; n (%) is shown for categorical variable.

A two-sample t-test was used for normally distributed variables; the Mann–Whitney U test was used for skewed variables; chi-square test was used for categorical variables.

eGFR: estimated glomerular filtration rate.

Definition: obesity: body mass index ≥ 25 kg/m^2^; hypercholesterolemia: non-high-density lipoprotein-C ≥ 170 mg/dL or take lipid medicine (non-high-density lipoprotein-C = total cholesterol − high-density lipoprotein-C).

In the overall population (n = 979), EP had significantly lower odds of AC presence than non-EP (Table 2). The OR was 0.62 (95%CI 0.39, 0.98) in the fully-adjusted model (Model 4), which adjusted for age, CT type, hypercholesterolemia, diabetes, hypertension, pack-year of smoking, ethanol, obesity, eGFR, CRP, and TG. Further controlling for creatine-adjusted urinary total ISFs (Model 5) based on a model already controlled for the major CVD risk factors (Model 3) did not materially change the estimate. Still, the result was no longer significant at 0.05 level (OR = 0.64, 95%CI 0.40, 1.01).

**Table 2:** The OR (95%CI) of aortic calcification presence for equol-producer vs. non- equol-producer (n = 979).

	Non-equol-producer (n = 487)	Equol-producer (n = 492)	
	OR	OR	95%CI
Model 1 (+ CT, age)	1.00 (ref)	0.57	0.37–0.88
Model 2 (Model 1 + HC, DM, HT)	1.00 (ref)	0.58	0.37–0.89
Model 3 (Model 2 + packyear, ethanol, obesity)	1.00 (ref)	0.61	0.39–0.96
Model 4 (Model 3 + CRP, TG, eGFR)	1.00 (ref)	0.62	0.39–0.98
Model 5 (Model 3 + ISFs)	1.00 (ref)	0.64	0.40–1.01

OR, odds ratio; CI, confidence interval; HT, hypertension; DM, diabetes; HC, hypercholesterolemia; eGFR, estimated glomerular filtration rate; TG, triglycerides; CRP, C-reactive protein; ISF, isoflavones.

ISFs = creatine-adjusted urinary levels of daidzein + genistein.

The R-square values for the models are: model 1, 0.51; model 2, 0.59; model 3, 0.62; model 4, 0.63; model 5, 0.63.

There was a marginally significant dose–response relationship across increasing equol amount categories. In the model that adjusted for hypercholesterolemia, diabetes, and hypertension (Model 2), the p-value for trend is 0.06 (Table 3). In the fully-adjusted model (Model 4), EP who had a low amount of creatinine-corrected urinary equol had significantly lower odds of AC presence than non-EP (OR = 0.51, 95% CI 0.30, 0.88). However, EP having a high amount of equol did not have significantly lower odds of AC presence than non-EP (OR = 0.75, 95% CI 0.43, 1.32). Finally, log-transformed ISFs concentrations were not significantly associated with AC presence (Table 4).

**Table 3:** The OR (95% CI) of aortic calcification presence for low or high levels of equol vs. non-equol-producer (n = 979).

	Non- equol-producer (n = 487)	Low-equol (n = 245)		High-equol (n = 247)		*p*-trend
	OR	OR	95%CI	OR	95%CI	
Model 1 (+ CT, age)	1.00 (ref)	0.50	0.30–0.83	0.66	0.39–1.12	0.06
Model 2 (Model 1 + HC, DM, HT)	1.00 (ref)	0.51	0.30–0.84	0.67	0.39–1.14	0.06
Model 3 (Model 2 + smoking, ethanol, obesity)	1.00 (ref)	0.51	0.30–0.87	0.75	0.43–1.30	0.16
Model 4 (Model 3 + CRP, TG, eGFR)	1.00 (ref)	0.51	0.30–0.88	0.75	0.43–1.32	0.18
Model 5 (Model 3 + creatine-adjusted urinary total ISFs)	1.00 (ref)	0.54	0.31–0.93	0.75	0.43–1.30	0.18

OR, odds ratio; CI, confidence interval; CT, computerized tomography; HT, hypertension; DM, diabetes; HC, hypercholesterolemia; eGFR, estimated glomerular filtration rate; TG, triglycerides; CRP, C-reactive protein; ISFs, isoflavones.

Median (25th–75th) of equol among low-equol group is 992 (279–2179); among high-equol group is 11,072 (6478–20,477).

**Table 4:** The OR (95%CI) of aortic calcification presence for ISFs (n = 979).

Models	OR	95% CI
Model 1 (+ CT, age)	1.04	0.69–1.42
Model 2 (Model 1 + HC, DM, HT)	1.07	0.77–1.50
Model 3 (Model 2 + smoking, ethanol, obesity, CRP, TG, eGFR)	1.18	0.82–1.69

OR, odds ratio; CI, confidence interval; CT, computerized tomography; HT, hypertension; DM, diabetes; HC, hypercholesterolemia; eGFR, estimated glomerular filtration rate; TG, triglycerides; CRP, C-reactive protein; ISFs, isoflavones.

ISFs = creatine-adjusted urinary levels of daidzein + genistein, and ISFs were log-transformed.

## Discussion

This population-based cross-sectional study among 979 Japanese men aged 40–79 years showed that people capable of producing equol, which consisted of half of the sample, had significantly lower odds of AC presence than those who did not produce equol. This association was independent of CVD risk factors. We observed a marginally significant dose–response relationship between equol and AC presence. ISFs were not significantly associated with AC presence. Our previous study found a non-significantly lower level of AC among EP than non-EP in a sample of 243 men aged 40–49 in Japan^19^. The present study expanded our previous findings with a larger effect size, sample size, and age range. The present study is the first to show that EP had a significantly lower atherosclerotic burden in the aorta than non-EP.

One cohort study and two cross-sectional studies in Asian populations supported the “equol hypothesis” that the health benefits of soy-based diets are more significant in EP than in non-EP^20^. A nested case–control study of CHD in 1130 Chinese women found a significant inverse association of incident CHD with equol (OR: 0.46, 95%CI 0.24, 0.89, comparing the lowest to highest quartile) but not with ISFs and their other metabolites^6^. Similar to the present study, we reported that equol-producing status, but not serum levels of ISFs, was significantly and inversely associated with prevalent CAC (OR: 0.1, 95%CI 0.01, 0.9) in Japanese men aged 40–49 years in ERA JUMP^21^. More recently, we have reported that EP had marginally lower AC scores in Japanese men (− 209, 95%CI − 455, 36) than non-EP^19^. However, secondary analyses of three RCTs of ISFs in Western populations reported no significant association of equol producing status with the progression of atherosclerosis^8,22,23^. The null results are very likely to be a lack of statistical power due to low rates of EP in Westerners^8,22^ and the short duration of intervention^22,23^.

We did not observe a significant dose–response relationship between equol amount and the odds of AC presence. Therefore, we performed a post-hoc exploratory analysis stratified by the age group because age was a strong determinant of AC presence: the OR of AC > 0 for a 1-year increase in age was 1.18 (95%CI 1.14, 1.21). We found that equol producing status was positively associated with markedly high odds of AC presence in the group of 70 + years (OR = 8.90, 95%CI 0.79, 99.99, Table S1). After excluding people aged 70 and over, the dose–response analysis showed significant and marginally significant trends in the odds of AC presence across increasing equol levels, supporting the dose–response relationship between equol and AC presence (Table S2). Reasons for the positive association in those aged 70 and over are unknown. Excluding people aged 70+ years, the significant inverse association between EP and AC presence became more pronounced (Table S3).

We also examined the association between equol producing status and the AC category (1–99, 100–299, 300–999, ≥ 1000, referencing AC = 0) to examine which AC category (lower or higher) was more strongly associated with equol-producing status or if there is a decreasing gradient of association from low AC score to high AC score. Across increasing AC categories, EP showed lower odds of having a higher AC category than non-EP (Table S4). The log odds of being in the AC category of 1 to 99 compared to no AC presence would decrease by 52% (95%CI 0.30, 0.79, Model 3) if moving from non-EP to EP after controlling for all confounders. In the model that controlled for traditional CVD risk factors only, the odds of being in AC category of 300 to 999 compared to no AC presence would decrease by 46% (95%CI 0.33, 0.91, Model 2) if a non-EP was an EP. We repeated the multinomial regression, excluding those aged 70 and over. We observed that the estimates of having each AC category were further away from null among people aged below 70 years than the estimates among the overall population (Table S5).

The strength of association between equol producing status and AC remained significant after adjusting for the traditional CVD risk factors such as diabetes, hypertension, obesity, and hyperlipidemia, suggesting that equol may benefit the cardiovascular system through other pathways. Our recent systematic review, through reviewing evidence on the cell, animals, and humans, found that these unique pathways may include antioxidation, anti-inflammation, vasorelaxation, and anti-calcification properties of equol^20^. Our review also supported that equol but not ISFs was significantly associated with atherosclerosis.

Although this study examined atherosclerosis, other vascular effects of equol have been reported. A crossover RCT of equol among overweight or obese subjects in Japan reported that supplementation of equol significantly improved arterial stiffness compared to the control group^24^. In the US and Japan, three RCTs of equol reported a significant reduction in vasomotor symptoms and hot flashes in peri- and post-menopausal women^25–27^. We recently reported that EP had significantly lower white matter lesion volume (WML) in the brain among cognitively normal older adults in Japan, a biomarker of cerebral small vessel disease compared to non-EP. Equol-producing status was determined 6–9 years before WML was measured. Serum levels of ISFs did not relate to WML^28^.

The ability to generate equol depends on the availability of the equol-producing gut microflora. The identified equol-producing bacterial strains are Adlercreutzia equolifaciens, Asaccharobacter celatus, Enterorhabdus mucosicola, Slackia isofla-voniconvertens, and Slackia equolifaciens^29–31^. The encoding of such bacterial strains involved key genes^11–13^. For example, the genes dzr, ddr and tdr, which code for daidzein reductase, dihydrodaidzein reductase and tetrahydrodaidzein reductase respectively, can be synthesized and cloned in E. coli clones harboring pUC57-equol^11^. Future findings will provide new knowledge on the metabolic transformation of daidzein into equol.

Our study has several limitations. First, we cannot establish causality due to the cross-sectional study design. Second, no optimal cut-off points for EP are established. However, different definitions of EP (urinary level of equol ≥ 20, ≥ 40, ≥ 60, ≥ 80) yielded similar results in our study (data not shown). Third, all the participants were men. Sex may modify the association between equol and AC presence. However, when SESSA was designed, prevalence of vascular calcification (i.e., CAC and AC) in women in the general Japanese population was expected to be very low^32,33^ and it is unethical to expose women to radiation. For this reason, women were not recruited to SESSA. Finally, our findings may be attributed to residual confounding. Our study has several strengths. First, this was a community-based study that utilized a random sampling method, thus, at least the results are generalizable to Japanese men in Japan. Second, compared to our previous analysis about equol producing status and AC score in the ERA JUMP study, the sample size in the current study was larger and the range of age of the participants was wider. Prior to the ERA JUMP and SESSA studies conducted by this research group, there had not been any studies on equol in men. Third, daidzein was detected in all the participants, and thus misclassification of equol producing status was unlikely. Fourth, AC, our outcome variable, predicts future CVD event as powerful as CAC, a well-established biomarker of coronary atherosclerosis^17,34–38^.

In conclusion, in Japanese men aged 40–79, EP had a significantly lower AC burden than non-producers while ISFs were not significantly associated with AC presence. Research on the association between equol-producing status and atherosclerosis is almost impossible in Western countries because of the minimal dietary intake of ISFs and the low rate of EP in Western countries^9^. Therefore, additional studies in East Asian countries with larger sample sizes for both sexes are warranted.

## Methods

### Study population and measurements

SESSA is an ongoing prospective, population-based study of a random sample from a general Japanese national^39^. Between 2006 and 2008, 2381 Japanese men 40 to 70 years of age were randomly selected based on age strata from Kusatsu City, Japan and sent an invitation to participate in the baseline survey of the SESSA study. Of these men, 1096 agreed to participate. The population of 40- to 79-year-old men in Kusatsu city was 25,394 in 2005. Therefore, the extraction rate was 9.4%, and the participation rate in this survey was 46%. Participants eligible for the present study were 1094 men, of which we excluded those with missing values in urinary equol (n = 2), AC score (n = 7), pack-year of smoking (n = 9), ethanol consumption (n = 20), and history of myocardial infarction or stroke (n = 1). We also excluded those with a history of myocardial infarction or stroke (n = 60), or with estimated glomerular filtration rate (eGFR) < 45 ml/min/1.73 m^2^ (n = 16) because they may not reflect accurate urinary equol^40^. A total of 979 participants were analyzed in the present study (Fig. S1). The study was approved by the Institutional Review Boards of Shiga University of Medical Science (Otsu, Japan) and the University of Pittsburgh (Pittsburgh, US). All participants provided written informed consent. All methods were performed in accordance with relevant guidelines and regulations.

A self-administered questionnaire was used to obtain information on demography, alcohol drinking, smoking habits, physical activity, medication use, etc. The frequency of alcohol consumption during a typical week and the total alcohol intake were determined^41^. Participants who smoked in the last 30 days were defined as current smokers, whereas participants who had never smoked before were defined as never smokers. Smokers were queried for the average number of cigarettes smoked each day. Bodyweight and height were measured in the physical examination, and BMI was calculated as weight (kg) divided by height squared (m^2^). Obesity was defined as BMI ≥ 25 kg/m^2^^42^. Blood pressure was measured twice consecutively of the seated participant after sitting quietly for 5 min, using an automated sphygmomanometer (BP-8800; Omron Health Care Co. Ltd, Tokyo, Japan). The average of the two measurements was used. Hypertension was defined as the use of antihypertensive medication, systolic blood pressure (SBP) ≥ 140 mmHg, or diastolic blood pressure ≥ 90 mmHg.

Blood specimens were obtained after a 12-h fast. Serum lipid concentrations were determined at a single laboratory (Shiga Laboratory; MEDIC, Shiga, Japan) certified for standardized lipid measurements according to the United States Centers for Disease Control and Prevention/Cholesterol Reference Method Laboratory Network. Total cholesterol and triglycerides (TG) were measured using enzymatic assays, and high-density lipoprotein cholesterol (HDL-C) was determined. Hypercholesterolemia was defined as non-HDL-C ≥ 170 mg/dL or the use of dyslipidemia medication. Non-HDL-C was the difference between total cholesterol (mg/dL) and HDL-C (mg/dL) and was a biomarker of dyslipidemia^43^. Plasma glucose was determined from NaF-treated plasma using a hexokinase glucose-6 phosphate-dehydrogenase enzymatic assay. Glycated hemoglobin A1C (HbA1c) was measured using latex agglutination immunoassays according to the protocol by the Japanese Diabetes Society and converted to the National Glycohemoglobin Standardization Program value. Diabetes was defined as either fasting glucose ≥ 126 mg/dL, HbA1c ≥ 6.5%, or medication use^44^. C-reactive protein (CRP) was measured by nephelometry using a BN II Analyzer^45^. Serum creatinine was measured using an enzymatic method (Espa CRE-liquid II; NIPRO, Osaka, Japan)^46^. We used the CKD Epidemiology Collaboration (CKD-EPI) equation modified for the Japanese^47^ to calculate eGFR.

### Measurement of urinary daidzein, genistein, and equol

The urine concentration of equol, daidzein, and genistein (LC Laboratories, Woburn, MA, USA) was measured at Saga Nutraceuticals Research Institute, Otsuka Pharmaceutical Co., Ltd. The sample preparation procedure was reported elsewhere^48^. In brief, (R,S)-equol-d4, daidzein-d6, and genistein-2′,3′,5′,6′-d4 (Toronto Research Chemicals, North York, Canada) as the internal standards were added to the urine sample and incubated with β-glucuronidase (Sigma-Aldrich, St Louis, MO, USA) for 1-h at 37 °C for enzymatic hydrolysis. The sample was cleaned up by Isolute SLE + 96-well plates (Biotage, Uppsala, Sweden), dried under nitrogen, and then reconstituted with 50% methanol. The sample was analyzed using liquid chromatography-tandem mass spectrometry (AB SCIEX Triple Quad 5500 + mass spectrometer) with atmospheric pressure chemical ionization ion source in the negative ion mode on the Shimadzu Nexera X2 UHPLC system. The multiple reaction monitoring transitions monitored were 241/121, 246/94, 253/223, 256/137, 269/133, 274/137 for equol, equol-d4, daidzein, daidzein-d6, genistein, and genistein-d4, respectively. The analytic coefficient of variation (CV) was 3.0–8.6% for equol, 3.6–12.0% for daidzein, and 3.5–10.3% for genistein. The lower detection limit for urinary equol, daidzein, and genistein are 4.13, 19.67, and 17.59 nmol/L. All participants' levels of these three markers were higher than the lower limit. The observer of the measurement of equol, daidzein, and genistein was blinded to the AC measurement.

### Measurement of AC

AC measurements have been described in detail elsewhere^49^. In brief, AC was measured using electron-beam computed tomographic (EBCT) with the C-150 scanner (Imatron, South San Francisco, CA, US) or 16-channel multi-detector-row computed tomography (MDCT) with the Aquilon scanner (Toshiba, Tokyo, Japan). AC lesions were considered to be present when three contiguous pixels (1 mm^2^) with an attenuation of ≥ 130 Hounsfield units (HU) were identified. Images were obtained from the aortic arch to the iliac bifurcation at every 6-mm slice for EBCT and 7-mm slice for MDCT to evaluate AC, with a scan time of 100 ms (EBCT) or 320 ms (MDCT). Agatston scores^50^ were obtained by multiplying the pixel area (mm^2^) by the density score depending on the highest density measurement (HU) anywhere in the plaque and summing all lesion scores. Readings of CT images were performed by trained physicians who were blinded to the clinical information as well as the equol measurement of the participants.

### Statistical analysis

Characteristics of participants were expressed as medians (25th–75th percentile) for continuous variables and as percentages for categorical variables by equol producing status. The difference in characteristics between equol-producers and non-producers was tested by the Kruskal–Wallis test when the variable was continuous and by the Chi-square test when the variable was categorical. Similar to what was proposed by Setchell et al*.*^51^, equol-producers in our study were defined as log10-transformed urinary equol to daidzein ratio of > − 1.5 (Fig. S2). The urinary equol to daidzein ratio provided a clearer distinction of equol-producer status than the absolute equol concentrations because it is independent of ISFs intake and minimizes interindividual variation in ISFs pharmacokinetics^51^. Since − 1.5 divides the distribution of the data into two peaks, if − 1.5 is used as a cut point, it will effectively separate those with a high ability to produce equol from those with a low ability (Fig. S2). AC presence was defined as AC score > 0^52–54^. In addition, creatine-adjusted urinary equol was defined as the ratio of urinary equol to urinary creatine. The same equation was applied to creatine-adjusted daidzein and genistein. We also categorized AC score into five groups: 0, 1–99, 100–299, 300–999, ≥ 1000.

The analytical methods were chosen based on a prior hypothesis. We used logistic regression to examine the association of equol producing status with the AC presence. Models were performed in the following orders: (1) adjusted for study design-related factor, CT type (EBCT vs. MDCT), and age, which is a strong indicator of atherosclerosis progression; (2) further adjusted for the key CVD risk factors (diabetes, hypercholesterolemia, and hypertension^55^); (3) further adjusted for other CVD risk factors such as pack-year of smoking, obesity, and ethanol; (4) further adjusted for CRP, TG, and eGFR; (5) further adjusted for creatine-adjusted urinary daidzein because we want to get the effect of equol independent of daidzein; (6) based on #4, further adjusted for ISFs. A *p*-value lower than 0.05 was considered statistically significant.

We performed logistic regression to evaluate the dose–response relationship between equol concentration categories and AC presence. We categorized participants into three groups: non-EP, low-equol concentration (creatine-adjusted equol amount < the median value of equol among EP), and high-equol concentration (≥ the median value of equol among EP). Confounders controlled in each model were the same as above. In addition, the association between log-transformed urinary adjusted total ISFs (daidzein + genistein) and AC presence was assessed by logistic regressions, adjusted for the same sets of confounders. All analyses were performed using R Statistical Software Version 4.0.2 (Foundation for Statistical Computing, Vienna, Austria).

## Supplementary Information

Supplementary Information.

## Data Availability

Data are available from the authors upon reasonable request and with permission of the Shiga Epidemiological Study of Subclinical Atherosclerosis (SESSA) (https://shiga-publichealth.jp/sessa/). For further information regarding data availability, please contact the corresponding author, Akira Sekikawa (akira@pitt.edu).
