# Supplementary material for: Cross-sectional association of equol producing status with aortic calcification in Japanese men aged 40–79 years
Source: Sci Rep. 2022 Nov 22;12:20114. doi: 10.1038/s41598-022-24659-8 (PMC9684435; doi:10.1038/s41598-022-24659-8)
Supplement: Supplementary file 1 — Supplementary Information. [file 41598_2022_24659_MOESM1_ESM.docx]

| Table S1. The ORs (95%CI) of AC presence (AC>0) for EP vs. non-EP in participants in different age groups (n=979) | | |
| --- | --- | --- |
| Age groups | OR | 95%CI |
| Age 40-49 (N=119) | 0.93 | 0.38 - 2.31 |
| Age 50-59 (N=189) | 0.28 | 0.12 - 0.64 |
| Age 60-69 (N=386) | 0.53 | 0.24 - 1.15 |
| Age 70-79 (N=285) | 8.90 | 0.79 - 99.99 |

OR, odds ratio; CI, confidence interval; EP, equol-producer

| Table S2. The ORs (95%CI) of AC presence (AC>0) for low or high amounts of equol vs. non-EP in men aged <70 years (n=694) | | | | | | | |  |
| --- | --- | --- | --- | --- | --- | --- | --- | --- |
|  | non-EP (n=487) | Low-equol (n=245) | | High-equol (n=247) | | p-trend | |  |
|  | OR | OR | 95%CI | OR | 95%CI | |  | |
| Model 1 (+ CT, age) | 1.00 (ref) | 0.45 | 0.27 - 0.75 | 0.57 | 0.33 – 0.98 | | 0.01 | |
| Model 2 (Model 1 + HC, DM, HT) | 1.00 (ref) | 0.46 | 0.26 - 0.77 | 0.63 | 0.35 - 1.12 | | 0.02 | |
| Model 3 (Model 2 + smoking, ethanol, obesity) | 1.00 (ref) | 0.45 | 0.26 - 0.78 | 0.63 | 0.35 - 1.12 | | 0.046 | |
| Model 4 (Model 3 + CRP, TG, eGFR) | 1.00 (ref) | 0.45 | 0.25 - 0.79 | 0.63 | 0.35 - 1.13 | | 0.053 | |
| Model 5 (Model 3 + creatine-adjusted urinary total ISFs) | 1.00 (ref) | 0.48 | 0.26 - 0.86 | 0.63 | 0.35 - 1.13 | | 0.06 | |
| OR, odds ratio; CI, confidence interval; CT, computerized tomography; HT, hypertension; DM, diabetes; HC, hypercholesterolemia; eGFR, estimated glomerular filtration rate; TG, triglycerides; CRP, C-reactive protein; ISFs, isoflavones | | | | | | | |  |
| Median (25th – 75th) of equol among low-equol group is 992 (279 - 2179); among high-equol group is 11072 (6478 - 20477). | | | | | | | |  |

| Table S3. The ORs (95%CI) of AC presence (AC>0) for EP vs. non-EP among people <70 years old (N=694) | | |
| --- | --- | --- |
|  | OR | 95%CI |
| Model 1 (+CT, age) | 0.49 | 0.31 - 0.77 |
| Model 2 (Model 1 + HC, DM, HT) | 0.50 | 0.31 - 0.78 |
| Model 3 (Model 2 + smoking, ethanol, obesity, CRP, TG, eGFR) | 0.50 | 0.31 - 0.81 |
| Model 4 (Model 3 + creatine-adjusted total ISFs) | 0.52 | 0.33 - 0.83 |

OR, odds ratio; CI, confidence interval; CT, computerized tomography; HT, hypertension; DM, diabetes; HC, hypercholesterolemia; eGFR, estimated glomerular filtration rate; TG, triglycerides; CRP, C-reactive protein; ISFs, isoflavones

| Table S4. The ORs (95%CI) of AC categories for EP vs. non-EP (n=979) | | | | |
| --- | --- | --- | --- | --- |
| Model | AC category | OR | 95%CI |  |
| Model 1 (+ CT, age) | 0 (n=162) | 1.00 (ref) |  |  |
|  | 1 to 99 (n=180) | 0.47 | 0.29 - 0.75 |  |
|  | 100 to 299 (n=139) | 0.73 | 0.43 - 1.22 |  |
|  | 300 to 999 (n=206) | 0.52 | 0.31 - 0.86 |  |
|  | ≥ 1000 (n=292) | 0.78 | 0.47 - 1.28 |  |
| Model 2 (Model 1 + HC, DM, HT) | 0 | 1.00 (ref) |  |  |
|  | 1 to 99 | 0.47 | 0.29 - 0.76 |  |
|  | 100 to 299 | 0.73 | 0.43 - 1.23 |  |
|  | 300 to 999 | 0.54 | 0.33 - 0.91 |  |
|  | ≥ 1000 | 0.81 | 0.48 - 1.35 |  |
| Model 3 (Model 2 + smoking, ethanol, obesity, CRP, TG, eGFR) | 0 | 1.00 (ref) |  |  |
|  | 1 to 99 | 0.48 | 0.30 - 0.79 |  |
|  | 100 to 299 | 0.82 | 0.48 - 1.40 |  |
|  | 300 to 999 | 0.61 | 0.36 - 1.04 |  |
|  | ≥ 1000 | 0.92 | 0.54 - 1.57 |  |

OR, odds ratio; CI, confidence interval; CT, computerized tomography; HT, hypertension; DM, diabetes; HC, hypercholesterolemia; eGFR, estimated glomerular filtration rate; TG, triglycerides; CRP, C-reactive protein; ISFs, isoflavones

| Table S5. The ORs (95%CI) of AC categories for EP vs. non-EP among people <70 years (n=694) | | | | |
| --- | --- | --- | --- | --- |
| Model | AC category | OR | 95%CI |  |
| Model 1 (+ CT, age) | 0 (n=162) | 1.00 (ref) |  |  |
|  | 1 to 99 (n=180) | 0.42 | 0.26 - 0.69 |  |
|  | 100 to 299 (n=139) | 0.65 | 0.37 - 1.14 |  |
|  | 300 to 999 (n=206) | 0.45 | 0.26 - 0.78 |  |
|  | ≥ 1000 (n=292) | 0.67 | 0.38 - 1.16 |  |
| Model 2 (Model 1 + HC, DM, HT) | 0 | 1.00 (ref) |  |  |
|  | 1 to 99 | 0.42 | 0.25 - 0.69 |  |
|  | 100 to 299 | 0.66 | 0.38 - 1.16 |  |
|  | 300 to 999 | 0.46 | 0.26 - 0.81 |  |
|  | ≥ 1000 | 0.70 | 0.39 - 1.23 |  |
| Model 3 (Model 2 + smoking, ethanol, obesity, CRP, TG, eGFR) | 0 | 1.00 (ref) |  |  |
|  | 1 to 99 | 0.44 | 0.26 - 0.73 |  |
|  | 100 to 299 | 0.72 | 0.40 - 1.29 |  |
|  | 300 to 999 | 0.50 | 0.28 – 0.91 |  |
|  | ≥ 1000 | 0.82 | 0.45 - 1.51 |  |

OR, odds ratio; CI, confidence interval; CT, computerized tomography; HT, hypertension; DM, diabetes; HC, hypercholesterolemia; eGFR, estimated glomerular filtration rate; TG, triglycerides; CRP, C-reactive protein; ISFs, isoflavones


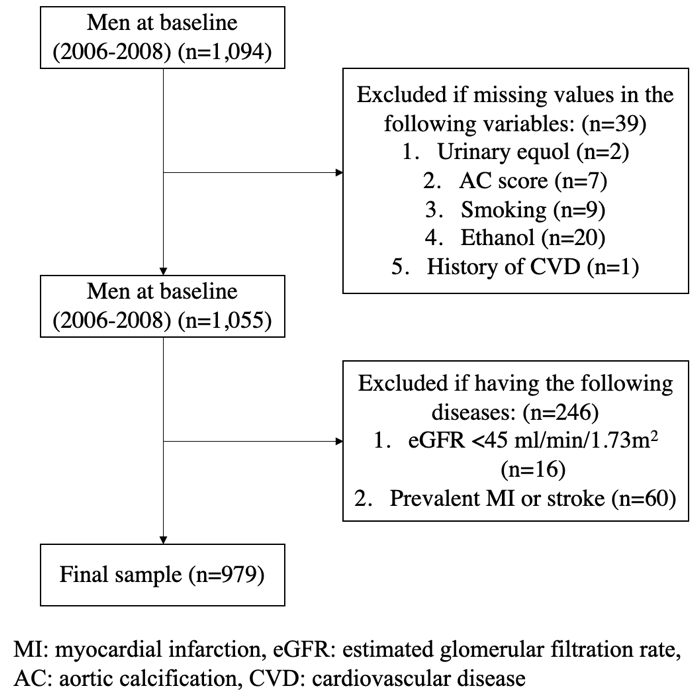


Figure S1. The flow-chart showing the sample size


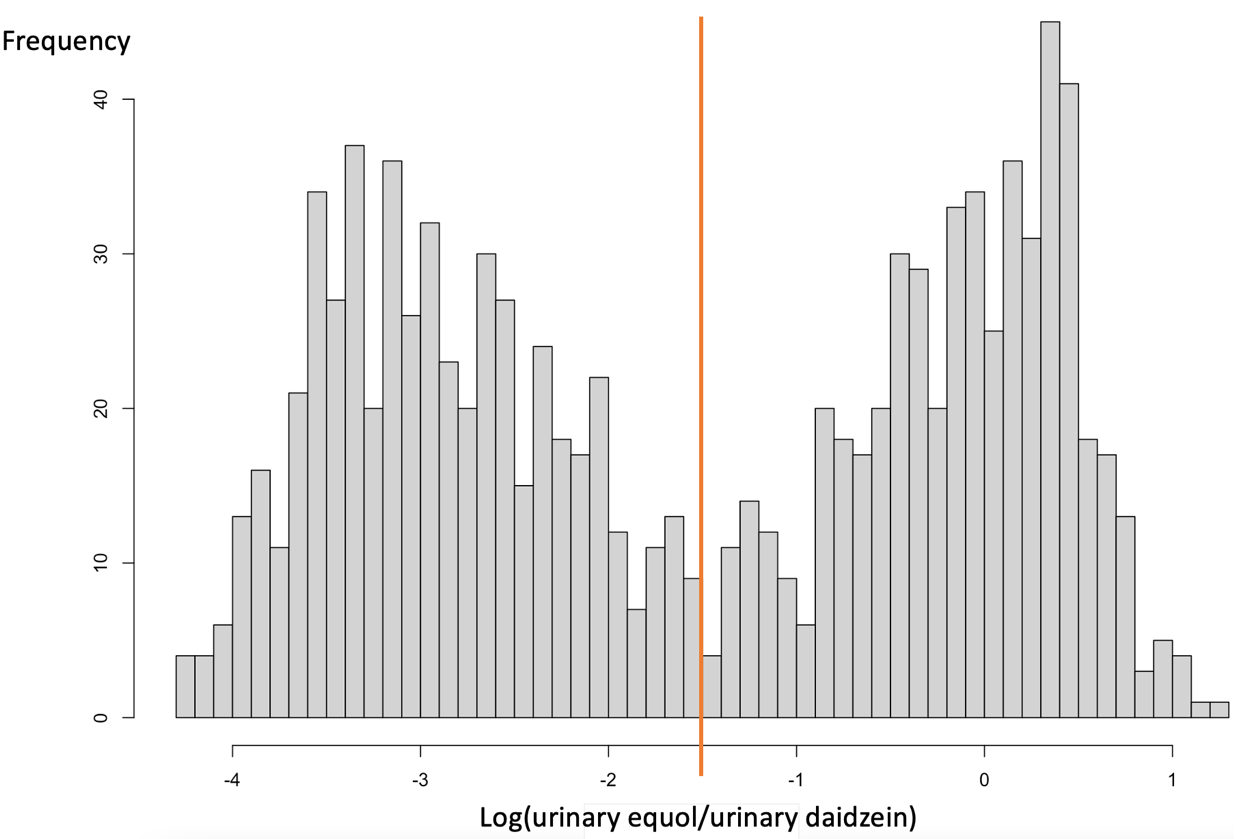


Figure S2. The distribution of log-transformed urinary equol/daidzein and the cut-off point chosen to define equol-producer
